# Silencing of juvenile hormone‐related genes through RNA interference leads to molt failure and high mortality in the spongy moth

**DOI:** 10.1111/1744-7917.70117

**Published:** 2025-07-14

**Authors:** Wenzhuai Ji, Qing Xie, Xiaoxiao Chang, Juan Shi

**Affiliations:** ^1^ Beijing Key Laboratory for Forest Pest Control and Sino‐French Joint Laboratory for Invasive Forest Pests in Eurasia, College of Forestry Beijing Forestry University Beijing China

**Keywords:** juvenile hormone acid methyltransferase (JHAMT), juvenile hormone epoxide hydrolase (JHEH), *Lymantria dispar*, pest control, RNAi

## Abstract

*Lymantria dispar* (spongy moth) is a globally polyphagous and destructive pest, with the larval stage being the main destructive stage. The larvae feed on more than 600 species of plants ranging from oaks to conifers, and they are capable of completely defoliating entire trees, leading to significant ecological and economic losses. Chemical insecticides are commonly used to control them, but these methods are expensive and nonspecific. Therefore, RNA interference (RNAi) is being explored as an alternative strategy to control spongy moth. Juvenile hormones (JHs) play a crucial role in regulating insect growth, diapause, and reproduction, making them potential targets for pest control. This study investigated whether 2 enzymes involved in the JH pathways—JH acid methyltransferase (JHAMT) from the biosynthetic pathway and JH epoxide hydrolase (JHEH) from the metabolic pathway—could be used for RNAi‐based control. *Ldjhamt* showed predominant expression during the larval stages and *Ldjheh* exhibited high expression throughout development. Both genes had the highest expression in the fat body. RNAi silencing using specific double‐stranded RNAs resulted in a significant decrease in the expression level of target genes, as well as a significant reduction in larval survival rate, pupation rate and eclosion rate. Additionally, many larvae and pupae died due to developmental abnormalities, and the adult size of the treated group was smaller than the control group. These findings demonstrate that *jhamt* and *jheh* are vital for the growth and development of spongy moths and suggest they could be effective targets for RNAi‐based pest control strategies.

## Graphical abstract

The feasibility of using RNA interference to control the globally important quarantine pest, the spongy moth. Targeting genes related to JHs play an important role in the growth and development of insects. First, the open reading frames (ORFs) of *Ldjhamt* and *Ldjheh* were identified and characterized, and the target genes were cloned and double‐stranded RNA was synthesized. Subsequently, the 4th instar larvae were injected, and the results showed that the injection led to a significant increase in the death of spongy moth and a significant decrease in the expression level of the target gene.

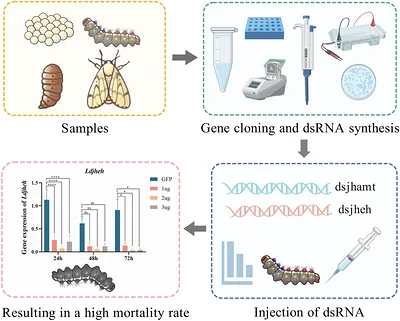

## Introduction

Foreign invasive alien species of forests (FIAS) pose a serious threat to global biodiversity. Research has proven that their impact on native plant species, community structure, and the environment has huge ecological and economic consequences (Sakai *et al.*, 2001; Srivastava *et al.*, 2020). *Lymantria dispar* L (Lepidoptera: Lymantriidae), widely distributed across much of the northern hemisphere, has been listed by the International Union for Conservation of Nature as one of the 100 most invasive alien species in the Global Invasive Species Database (Luque *et al.*, 2014; Srivastava *et al.*, 2020). The spongy moth, originally native to the Palearctic, later invaded North America via human hybridization experiments. The North American Plant Protection Organization considers the spongy moth an important quarantine pest (Forbush & Femald, 1896; Pogue & Schaefer, 2007). The species exhibits considerable biological significance in both its native and introduced ranges, especially as a destructive forest pest and invasive species (Inoue *et al.*, 2019). Specifically, the infestation is primarily in the larval stage and has a wide host range. In the early larval stages (1st to 3rd instar), larvae feed on the leaves of their host plants during the day and remain beneath the leaves at night (Leonard, 1974). In contrast, late‐instar larvae (4th to 6th instar) typically move both day and night and often abandon the trees they have been feeding on to migrate to new trees for feeding (Forbush & Fernald, 1896). This behavior results in late‐instar larvae having a wider range of host plants compared to early instar larvae, leading to greater damage (Barbosa, 1978). In severe cases, heavy feeding by the larvae leads to complete defoliation of the tree, resulting in significant damage and economic loss (Wallner, 1996). The European spongy moth, which is distributed in Europe and North America, has a host range of roughly 300 species, mainly concentrated in broadleaf trees (Pogue & Schaefer, 2007; Keena & Richards, 2020). In North America, the spongy moth poses a highly destructive threat to broadleaf trees and is estimated to cause economic losses of up to $3.2 billion per year (Bradshaw *et al.*, 2016). The Asian spongy moth, which is distributed in Asia, has a much wider host range and is capable of damaging about 600 species of plants. Its host plants include not only a wide range of broadleaf species, but also coniferous species and some agricultural crops (Liebhold, 1995; Baranchikov *et al.*, 1998). For example, as of 2018 it affected over 40 000 hectares of crops in Tongliao, causing huge economic losses (Bai *et al.*, 2020).

Due to the strong destructive power of the spongy moth and its ecological and economic impacts, chemical control is the main means to manage spongy moths in the fields (Liebhold & McManus, 1999). Since the 20th century, a variety of insecticides have been widely used in the prevention and control of spongy moths. Unfortunately, the frequent and widespread use of pesticides has adversely affected biodiversity and accelerates the development of insecticide resistance in insects. Typically, insects utilize their detoxification mechanism to deal with toxic plant chemicals and pesticides (Xu *et al.*, 2023). The insect population control strategies currently available involve using sex pheromones to interfere with and interrupt mating and viral or bacterial pathogen‐based “biocides” to kill the larvae (Mayo *et al.*, 2003; Whittle *et al.*, 2008; Harrison *et al.*, 2016). However, the sex pheromone disruption method is only effective at the initial stages of pest occurrence and is less effective when the population densities are high. Although nucleopoly hidrosis virus (NPV) exhibits high specificity and is harmless to humans and animals, and can be effective even at high population densities, its high production cost and limited market availability make it relatively costly to use in spongy moth control (Solter & Hajek, 2009). In addition, it can only be produced by infecting live spongy moth larvae, resulting in limited production (Boukouvala *et al.*, 2022). Despite the success of the product through improved technology, availability is still limited due to it being very labor‐intensive and costly to manufacture. Therefore, it is only used in treatment operations sponsored by the Forest Service (Coleman & Liebhold, 2023). Given the limitations of traditional control methods, researchers are actively exploring new alternative strategies, with biopesticides based on RNA interference (RNAi) being particularly notable.

In 1998, Andrew Fire and Craig Mello first demonstrated that double‐stranded RNAs (dsRNA) specifically and efficiently silence messenger RNAs (mRNAs) in organisms (Fire *et al.*, 1998). Since then, RNAi technology, based on dsRNA or small interfering RNA (siRNA), has been widely used to assess gene functions in fungi, plants, animals, and other eukaryotic organisms (Baulcombe, 2004; Labreuche & Warr, 2013; Rosa *et al.*, 2018; Koch & Wassenegger, 2021). RNAi, a post‐transcriptional gene silencing mechanism, is initiated by introducing dsRNA into the cell (Hannon, 2002). In insects, RNAi plays a crucial role in various biological processes (Fujita *et al.*, 2006; Hossain *et al.*, 2008; Zhou *et al.*, 2008; Irles *et al.*, 2013; Zhang *et al.*, 2024; Wang *et al.*, 2025). Thus, RNAi has demonstrated high efficiency in pest control. In this approach, insect‐specific RNAi biopesticides significantly inhibit the expression of the target genes, adversely affecting the pests and opening up the possibility of developing pest‐specific and sustainable management strategies (Baum *et al.*, 2007; Zhou *et al.*, 2008; Burand & Hunter, 2013; Gong *et al.*, 2013; Guo *et al.*, 2015; Li *et al.*, 2015; Peng *et al.*, 2020; Jain *et al.*, 2021; Li *et al.*, 2021; Hoang *et al.*, 2022; Ma *et al.*, 2023; Liu *et al.*, 2025). In addition, plant‐mediated RNAi insect‐resistant crops serve as a novel approach to control important agricultural pests; here, plants are genetically engineered to express dsRNA. Studies have proven that RNAi crops effectively inhibit the growth and multiplication of pests (Baum *et al.*, 2007; Head *et al.*, 2017). Thus, while traditional chemical insecticides cause damage to non‐target organisms and promote pesticide resistance, RNAi‐based methods are highly specific and significantly reduce the risk of harm to off‐target and non‐target species (Wang *et al.*, 2023), providing long‐term protection (de Andrade & Hunter, 2016; Sharma *et al.*, 2023).

The selection of target genes in RNAi experiments constitutes a critical step. Studies using insects’ own hormones and their metabolic enzymes can be used as a molecular biocontrol tool, which can lead to affecting the normal growth and development of the host (Swetha 2015). From this point of view, juvenile hormone (JH) metabolism has been targeted as a biological control strategy. JHs are sesquiterpene hormones essential for the regulation of molting, metamorphosis, reproduction, and stunting in insects (Richards, 1981; Hartfelder, 2000; Riddiford, 2012). Consequently, targeted disruption of its biosynthetic pathways or degradation mechanisms has been consistently recognized as a promising strategy for developing alternative insecticides characterized by low toxicity toward non‐target organisms (Minakuchi & Riddiford, 2006). A homeostatic balance of JHs is crucial for insects throughout their life cycle, and a combination of JH synthesis and metabolic processes helps maintain this balance. Typically, the biosynthesis of JHs involves the following 5 steps: 3 units of acetyl‐CoA combine to form mevalonate; mevalonate then gets converted to farnesyl pyrophosphate (FPP); FPP further gets transformed into farnesal by pyrophosphatase and dehydrogenated to farnesoic acid (FA) under the action of farnitol dehydrogenase; finally, FA gets catalyzed to JH by JH acid methyl transferase (JHAMT) and P450 epoxidase CYP15 (Bellés *et al.*, 2005). Among the various enzymes, JHAMT plays a key role in JH biosynthesis (Mayoral *et al.*, 2009); as a rate‐limiting enzyme in the JH synthesis pathway, it has been shown to play a crucial role in insect metamorphosis and reproduction (Kinjoh *et al.*, 2007; Daimon & Shinoda, 2013). Several studies have shown that inhibiting *jhamt* expression in insects via gene knockout or downregulation triggers unfavorable phenotypes, such as growth retardation, reduced reproductive capacity and stunting (Minakuchi *et al.*, 2008; Yin *et al.*, 2020; Tian *et al.*, 2021). Therefore, *jhamt* is considered an important target for the development of novel insect growth regulators or insecticides (Cusson *et al.*, 2013; Hiruma & Kaneko, 2013). Meanwhile, the metabolism of JH involves the synergistic action of several enzymes, including JH esterase (JHE), JH epoxide hydrolase (JHEH), and JH diol kinase (JHDK) (Hammock & Sparks, 1977; Li *et al.*, 2005; Seino *et al.*, 2010). Among these, the gene encoding JHE is one of the most widely studied. Additionally, recent studies have shown that JHEH plays a key role in the metabolism of JHs. It converts the juvenile hormone to JHD and further metabolizes the juvenile hormone acid to JHAD. It regulates insect growth, development, reproduction and metabolism (Mackert *et al.*, 2010; Tsubota *et al.*, 2010; Zhao *et al.*, 2017; Wen *et al.*, 2018). JHEH converts the juvenile hormone to JHD and further metabolizes the juvenile hormone acid to JHAD. The full‐length sequence of the *jheh* of the spongy moth has been successfully cloned and synthesized into dsRNA, and its effect on larvae has been detected by feeding methods. However, due to the rapid degradation of dsRNA in Lepidoptera, the efficiency of dsRNA delivered via the feeding route is lower, which results in low levels of gene silencing (Wen *et al.*, 2018).

Therefore, the present study aimed to evaluate the potential of RNAi targeting *jhamt* related to JH synthesis and JHE related to metabolism as a novel insecticide against the spongy moth. We screened *jhamt* and *jheh* genes from the spongy moth transcriptome database and conducted detailed bioinformatics analysis. Second, we assessed their stage‐specific and tissue‐specific expression profiles in the spongy moth. Finally, we synthesized dsRNA targeting these 2 genes and systematically studied their regulatory effects by injecting dsRNAs into the 4th instar larvae of spongy moths, based on the strong harmfulness of late‐instar larvae (4th–6th instars). We used 3 concentrations and time gradients in this experiment to identify the optimal concentration and time of RNAi. During the experiment, we evaluated the main growth and development parameters, survival rate, pupation rate and eclosion rate of spongy moth larvae, and calculated the developmental time at different stages to determine the function of target genes. The study's findings will deepen our understanding of the roles of *jhamt* and *jheh* in spongy moths and enhance the potential application of these 2 genes in pest management.

## Materials and methods

### Insect rearing

We collected egg masses from Zixia Mountain (30°33′N, 104°21′E), Chengdu, Sichuan Province, China, placed them in an insect feeding box and reared them on artificial diets at the Quarantine Laboratory of the Beijing Forestry University under the following conditions: 25 ± 1 °C temperature, 60%–70% humidity, and 16 h : 8 h (light : dark) photoperiod. Based on a previous report (Wang *et al.*, 2021), 5 larvae were maintained in each insect feeding box, and fresh feed was provided every 2 days to ensure adequate nutrition for the larvae.

### Cloning of Ldjhamt and Ldjheh

Primers (*jhamt*‐F/*jhamt*‐R and *jheh*‐F/*jheh*‐R) targeting *Ldjhamt* and *Ldjheh* were designed using DNAMAN9 software (version no. 10.0.0.2.100) and sent to and synthesized at the Beijing Qingke Biotechnology Co., Ltd; the primer sequences are listed in Table 1. Polymerase chain reaction (PCR) amplification was performed using 2 × Phanta Max Master Mix (Dye Plus) (P525‐01; Vazyme, Nanjing, China); Each reaction is carried out in a volume of 50 *µ*L, including 25 *µ*L of 2 × Phanta Max Master Mix, 2 *µ*L of each primer pair, 2 *µ*L of complementary DNA (cDNA) template and 19 *µ*L of ddH_2_O. The amplification procedure was as follows: 3 min at 95 °C, followed by 15 s at 95 °C, 15 s at 60 °C for 34 cycles, then 5 min at 72 °C, and finally maintained at 4 °C. PCR products were electrophoretically verified in 1% agarose gel (voltage 110 V, 30 min, 1 × Tris‐acetate – ethylenediaminetetraacetic acid as electrophoresis buffer). Samples with bright bands were selected and subjected to gel recovery using the TIANgel Purification Kit (DP219‐02; TianGen, Beijing, China). The purified product was mixed with the PEASY‐Blunt3 (CB111–01; TransGen Biotech, Beijing, China) vector and incubated at 25 °C for 30 min, then it was transformed into *Trans*5*α* Chemically Competent Cell (CD201‐01, TransGen Biotech, Beijing, China). Finally, the positive clones were sequenced at Qingke Biotechnology Co., Ltd and confirmed.

**Table 1:** Primers used in this study. T7 sequences in the RNA interference primers are underlined

Primers	Sequence (5′–3′)
*Jhamt*‐F	GCCGCAGAAGAACAAGTGTAAC
*Jhamt*‐R	CAAACATAGTGCTTCTCTGTAG
*Jheh*‐F	CTCTTTCACAGCTTTGAACACA
*Jheh*‐R	ATTCAAAGGTGGACGTGGAAAT
*Actin*‐F	AGAAGCACTTGCGGTGGACAAT
*Actin*‐R	ACCTGTACGCCAACACTGTCAT
*Jhamt*‐qPCR‐F	TCTCGGCTGTGACATAAA
*Jhamt*‐qPCR‐R	TTCACCACCTGTCTCCAT
*Jheh*‐qPCR‐F	TTCAGAGGCACCAACACG
*Jheh*‐qPCR‐R	CTCCAGCCACAAATGACG
ds*jhamt*‐F	TCACATTTCTCGGCTGTGAC
ds*jhamt*‐R	CGGCCTTCAAAGTGTTTTTC
ds*jhamt*‐t7‐F	TAATACGACTCACTATAGGGTCACATTTCTCGGCTGTGAC
ds*jhamt*‐t7‐R	TAATACGACTCACTATAGGGCGGCCTTCAAAGTGTTTTTC
ds*jheh*‐F	ATTCTGCTTGGACGTATGGC
ds*jheh*‐R	CCAGGAAGACTTGGTGCAAT
ds*jheh*‐t7‐F	TAATACGACTCACTATAGGGATTCTGCTTGGACGTATGGC
ds*jheh*‐t7‐R	TAATACGACTCACTATAGGGCCAGGAAGACTTGGTGCAAT

### Analysis of physicochemical properties of target proteins

The molecular weight, isoelectric point (pI), and amino acid composition of the deduced protein sequences were determined using Expasy (http://web.expasy.org/protparam/). National Center for Biotechnology Information Domain Database (https://www.ncbi.nlm.nih.gov/Structure/cdd/wrpsb.cgi) was used to confirm conserved structural domains. SignalP (SignalP 4.1‐DTU Health Tech‐Bioinformatic Services) was used to predict the signal peptides of the proteins, and TMHMM 2.0 (http://www.cbs.dtu.dk/services/TMHMM) was used to predict the transmembrane structural domains. Further, SOPMA (https://npsa.lyon.inserm.fr/cgi‐bin/npsa_automat.pl?page=/NPSA/npsa_sopma.html) and SWISS‐MODEL (https://swissmodel.expasy.org/) were used to predict the secondary and tertiary structures, respectively.

### Analysis of Ldjhamt and Ldjheh expression profiles

The expression profiles of the 2 key genes in *L. dispar* were measured at several developmental stages, including the egg, 1st to 6th instar larvae, pupae, and adult, to assess their expression profiles during development. The extraction of total RNA was completed using MolPure^®^ Cell/Issue Total RNA kit (18601ES59, Yeasen, Shanghai, China). In the selection of sample size, 200 eggs, 50 larvae of 1st–2nd instars each, 50 larva of 3rd–6th instars each, and 30 female and 30 male individuals were collected from pupa and adult, respectively. There were 5 sets of biological replicates using the above samples as a group. Finally, NanoDrop 8000 (Thermo, Waltham, MA, USA) and agarose gel electrophoresis were used to test purity, concentration, and integrity. Next, the Hifair^®^ II 1st Strand cDNA Synthesis Kit (11119ES60; YeaSen, Shanghai, China) was used to synthesize cDNA from 1 *µ*g of total RNA. In addition, to understand the tissue‐specific expression patterns, 4 key tissue samples (midgut, head, fat body and epidermis) were collected from a group of 50 5th instar larvae, with 5 sets of biological replicates. Finally, the dissected tissues were immersed in lysis buffer and total RNA was subsequently extracted.

Total RNA was extracted and cDNA synthesized using the method described in the previous paragraph and the obtained cDNA was diluted with ddH_2_O at a ratio of 1: 10. Specific primers were designed using Primer5 and sent to Qingke Biotechnology Co., Ltd for synthesis. Real‐time fluorescence quantitative PCR (RT‐qPCR) was performed with Hieff^™^ qPCR SYBR^®^ Green Master Mix (11201ES03, Yeasen, Shanghai, China) in a 20 *µ*L reaction mixture on a CFX Connec Real‐Time PCR System (Bio‐Rad, CA, USA) to detect the transcript levels of *Ldjhamt* and *Ldjheh*. The primer sequences of the target genes and the internal reference genes (*Actin*‐F, *Actin*‐R) used for qPCR are listed in Table 1.

The RT‐qPCR mixture was set as follows: 10 *µ*L of Hieff^™^ qPCR SYBR^®^ Green Master Mix, 0.4 *µ*L each of the upstream and downstream primers (10 *µ*mol/L), 1 *µ*L of template cDNA and 8.2 *µ*L of ddH_2_O. The amplification was carried out with the set program: initial denaturation at 95 °C for 1 min, followed by 40 cycles of denaturation at 95 °C for 10 s and annealing at 60 °C for 20 s. To ensure the reliability of the data, 3 biological and 3 technical replicates were conducted for each sample. Finally, the relative expression levels of the target genes were calculated based on the 2^−ΔΔCT^ method.

### Synthesis of dsRNA for the target gene

The dsRNA was prepared using the Megascript RNAi kit (AM1626, Ambion, Austin, TX, USA), following the manufacturer's instructions. Primers were designed for the target genes using the online tool SnapDragon (https://www.flyrnai.org/cgi‐bin/RNAi_find_primers.pl) and used for subsequent amplification after addition of the T7 promoter sequence at the 5′ end (Table 1). *GFP* exogenous gene fragments were used as controls. The amplicons were separated by agarose gel electrophoresis and purified as templates to synthesize the dsRNA. The positive and antisense strands were first synthesized from the amplicons under the catalysis of the T7 RNA polymerase and subsequently incubated at 37 °C for 5 h to promote annealing and form the dsRNA for the 2 target genes. Similarly, dsRNA was synthesized for the *GFP* gene as a control. The synthesized dsRNA was purified by isopropanol precipitation, dissolved in diethyl pyrocarbonate water, and stored at −80 °C to maintain stability. The purity of the synthesized dsRNA was analyzed using agarose gel electrophoresis, and its concentration was accurately determined by NanoDrop 8000, for further use in gene silencing.

### RNAi

RNAi was performed to silence *Ldjhamt* and *Ldjheh* genes using an injection method with a micro‐sampler (Hamilton, Bonaduz, Switzerland). In terms of selecting experimental subjects, we selected synchronously hatched larvae from 5 eggs and placed them in a constant temperature incubator for artificial feeding until the larvae developed to the 4th instar. Subsequently, we selected larvae of similar weight as experimental subjects to ensure consistency in experimental conditions. The dsRNA targeting *Ldjhamt* or *Ldjheh* was diluted with RNase‐free water to 1, 2, and 3 *µ*g/*µ*L concentrations and injected into the 4th instar larvae of the spongy moth. About 1 *µ*L of dsRNA was injected into each larva, and for each treatment, 3 biological and 3 technical replicates were carried out and 27 larvae were selected for each biological replication. The dsRNA was injected at the penultimate segment of the larval abdomen as close to the body wall as possible. The larvae of the negative control group were injected with an equal dose of ds*GFP*. The injected larvae were then transferred to insect feeding boxes and maintained under the following conditions: 25 ± 1 °C temperature, 60%–70% humidity, and 16 h : 8 h (light : dark) photoperiod. Then, the survival rate and molting of the larvae were monitored daily. During this period, injected larvae were individually reared in insect rearing boxes, each provided with 10 g of artificial feed. To keep the rearing environment clean, feces were uniformly cleaned and replaced with fresh feed every 2 days. Total RNA was extracted from larvae randomly selected after 24, 48, and 72 h of treatment and reverse transcribed to cDNA (3 biological replicates and 3 technical replicates per treatment). RT‐qPCR was performed using the cDNA as a template to determine the silencing efficiency of *Ldjhamt* and *Ldjheh* at 24, 48, and 72 h post‐injection. Finally, the relative expression levels of *Ldjhamt* and *Ldjheh* were assessed using a relative quantification method (2^−∆∆CT^). The remaining larvae injected with dsRNA were maintained under the same conditions until the adult stage. During this period, the mortality rate of larvae, changes in body length, weight, developmental time, pupation rate and adult emergence rate were continuously recorded as key indicators of growth and development.

### Data analysis

GraphPad was used to analyze the data and graph plotting. A one‐way analysis of variance (ANOVA) was applied to assess the difference between the treatment group (*Ldjhamt* and *Ldjheh* genes) and the negative control (ds*GFP*). The differences were considered statistically significant at *P ≤ 0.05; **P ≤ 0.01; ***P ≤ 0.001; ****P ≤ 0.0001.

## Results

### Physicochemical properties of Ldjhamt and Ldjheh proteins

Initially, we cloned the full‐length sequence of *Ldjhamt* (1 242 bp) gene with an ORF of 813 bp, encoding a protein with 271 amino acids. The molecular weight of its corresponding protein was predicted to be approximately 32 179.69 Da, and the theoretical pI value was predicted to be 5.38. Further, ProParam deduced the protein molecular formula as C_1466_H_2193_N_365_O_421_S_16_. Phe (F) and Lys (K) occupied 7.8% and 9.6% of the protein sequence. The average hydrophilicity index of the *Ld*JHAMT protein was −0.510, and the extinction coefficient was 55 600. Its fat coefficient was 71.81, and its instability coefficient was 30.27, which collectively indicated the stability of the protein. The TMHMM 2.0 and SignalP tools predicted that *Ld*JHAMT did not contain signal peptides and transmembrane regions and was considered a non‐secretory protein, this indicates that dsRNA can be effectively targeted to mRNA in the cytoplasm through injection delivery. The Wolf PSORT tool predicted cytoplasm localization for the protein. Furthermore, the SOPMA program on the PRABI website predicted 38.15%, 49.26%, 12.59%, and 0% of irregular coil, *α*‐helix, extended chain, and *β*‐fold, respectively, in the secondary structure of the *Ld*JHAMT protein. Subsequently, tertiary structure homology modeling in the SWISS‐MODEL platform built the tertiary structure of *Ld*JHAMT using the crystal structure of the JHAMT as a template. This approach indicated a high degree of homology between *Ld*JHAMT and the selected JHAMT model, thus validating the homology of the selected gene, providing structural biology evidence for the effectiveness of RNAi.

Similarly, we found that the ORF of *Ldjheh* was 1 458 bp long and encodes a protein with 486 amino acid residues. This protein was predicted to have a molecular weight of approximately 56 187.71 Da and a pI of 8.15. The molecular formula of the protein was deduced to be C_2613_H_3937_N_641_O_713_S_14_. In this protein, the Ile (I) and Phe (F) content was 7.4%. The total average hydrophilicity of the protein was −0.212, and the extinction coefficient was 103 375. Further, the fat coefficient was 83.42, and the instability coefficient was 37.32, indicating the stability of the protein. NetNGlyc 1.0 Server and TMHMM 2.0 predicted the presence of a transmembrane region in the protein. In addition, SignalP predicted that the protein does not possess a signal peptide, and Wolf PSORT predicted plasma membrane localization for the protein. This indicates that it may be involved in membrane‐bound metabolism processes; therefore, in the dsRNA design process, it is necessary to cover the functional domains exposed in the transmembrane region to ensure its effectiveness. A structural domain belonging to the epoxide hydrolase superfamily‐EHN, which exhibits the typical features of epoxide hydrolases, was found in the *Ld*JHEH protein from position 87 to 198. Furthermore, the SOPMA program available on the PRABI website predicted 50%, 37.86%, 12.14%, and 0% irregularly coiled, *α*‐helix, extended chain, and *β*‐fold in the protein, respectively. Finally, using the epoxide hydrolase of the larvae‐juvenile hormone of the silkworm, *Bombyx mori*, as a template, we predicted the tertiary structure of *Ld*JHEH. The approach predicted 48.92% sequence similarity, indicating that *Ld*JHEH is a homolog of JHEH. This proves that the target gene is involved in the molecular mechanism of JH metabolism, supporting the phenotype interpretation of subsequent RNAi experiments.

### Temporal and spatial expression patterns of Ldjhamt and Ldjheh

The expression patterns of *Ldjhamt* and *Ldjheh* at different developmental stages and in different tissues were systematically analyzed by RT‐qPCR. The analysis revealed that the 2 genes were expressed to different degrees at various developmental stages from egg to adult. The relative expression of *Ldjhamt* was significantly lower in the egg of the spongy moth while it reached a maximum in the 5th instar larvae; and then the expression decreased in the 6th instar stage (Fig. 1A). Notably, a sharp decrease in the expression of the *Ldjhamt* was detected in the pupal and adult stages. *Ldjhamt* expression was significantly higher in the larva than in the pupa and adult (*P* < 0.05). On the other hand, *Ldjheh* expression was the highest in the larvae at the 6th instar stage, followed by the 5th instar stage; the expression gradually decreased at the pupal and adult stages (Fig. 1B). These observations suggest that *Ldjheh* is highly expressed in the mature larva. Subsequent analysis of the various tissues of the larvae (fat body, epidermis, head, and midgut) showed that *Ldjhamt* and *Ldjheh* were expressed in all tissues. *Ldjhamt* showed the highest expression in the fat body, followed by the epidermis; meanwhile, the expression levels in the midgut and head tissues were relatively low (Fig. 1C). *Ldjheh* also showed the highest expression in the fat body, followed by the midgut. The expression of *Ldjheh* in these 2 tissues was significantly higher than that in the epidermis and the head; its expression was lowest in the head (Fig. 1D).

**Fig. 1:**
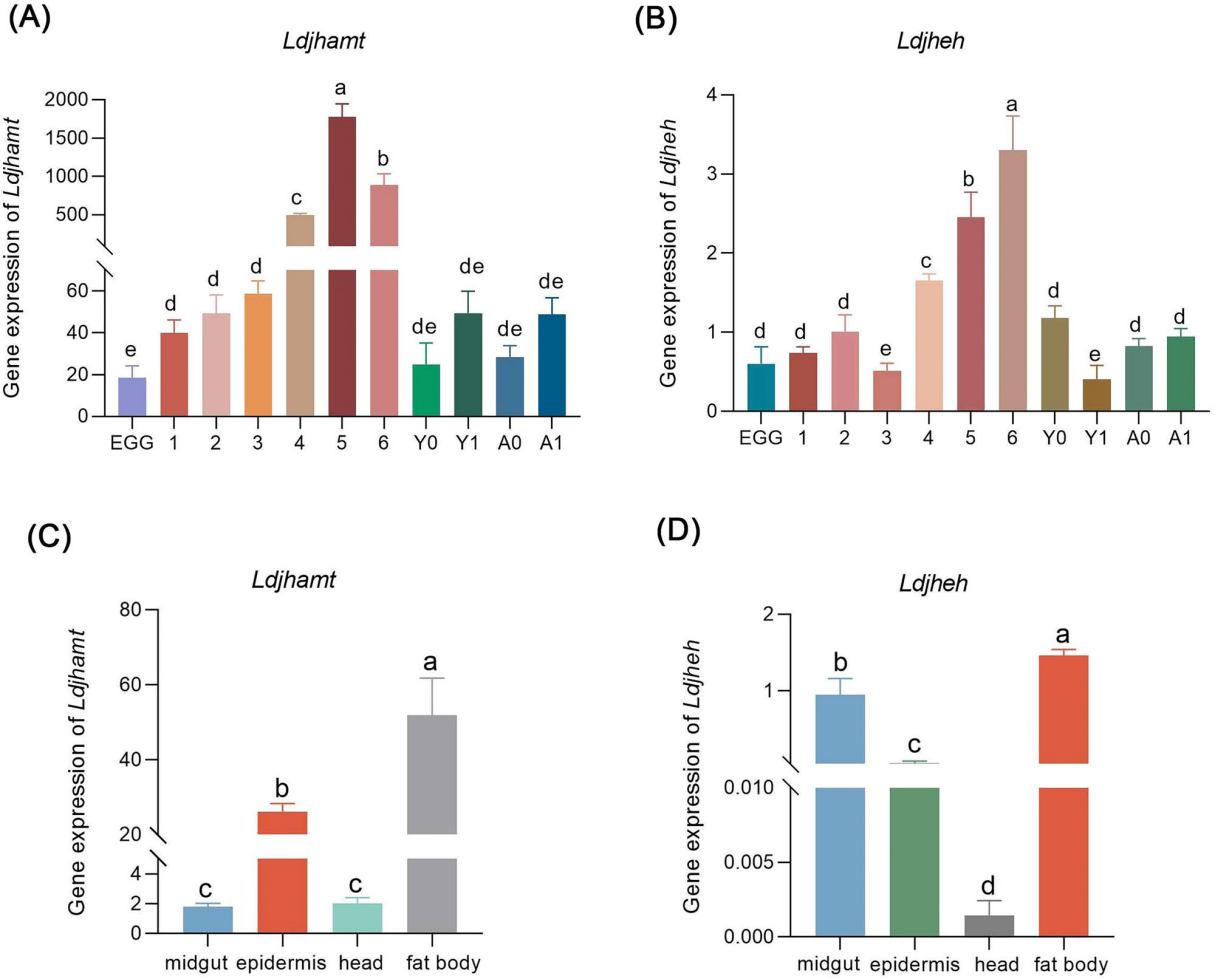
Expression of *Ldjhamt* and *Ldjheh* at different developmental stages and in different tissues of spongy moth. Data are expressed as the mean ± standard error from 3 independent biological replicates and lowercase letters indicate significant differences between treatments (*P *< 0.05, analysis of variance). Expression of (A) *Ldjhamt* and (B) *Ldjheh* at different developmental stages (1–6: 1–6 instar larvae; Y0: female pupae; Y1: male pupae; A0: female adult; A1: male adult). Expression of (C) *Ldjhamt* and (D) *Ldjheh* in different tissues.

### Effect of dsRNA injection on Ldjhamt and Ldjheh expression

We further investigated the effect of dsRNA injection on the mRNA levels of *Ldjhamt* and *Ldjheh* in the spongy moth larva. *Ldjhamt* and *Ldjheh* mRNA levels were significantly suppressed in the spongy moth larvae injected with the specific dsRNA compared with those injected with ds*GFP*. The level of inhibition in gene expression was closely related to the concentration of dsRNA injected and the time elapsed after injection. Specifically, 24 h after injection, 1, 2, and 3 *µ*g of dsRNA resulted in 65%, 85%, and 89% drop in *Ldjhamt* mRNA levels, respectively. At 48 h post‐injection, the *Ldjhamt* mRNA level decreased in the groups injected with 1 and 2 *µ*g dsRNA (74% and 73% inhibition, respectively). Meanwhile, the inhibition level of the experimental group injected with 3 *µ*g dsRNA decreased to 39%. After 72 h of injection, although the silencing efficiency decreased, the interference efficiency was still significant compared to the control group: the interference efficiencies of 1, 2, and 3 *µ*g were 59%, 82%, and 49%, respectively (Fig. 2A). Overall, for *Ldjhamt*, injection of 2 *µ*g dsRNA showed the best effect, while injection of 1 *µ*g showed a trend of first decreasing and then increasing inhibition levels. Although 3 *µ*g had the best interference effect within 24 h, the interference efficiency gradually decreased at 48 and 72 h, leading to a rebound in target gene expression levels (Fig. 2B).

**Fig. 2:**
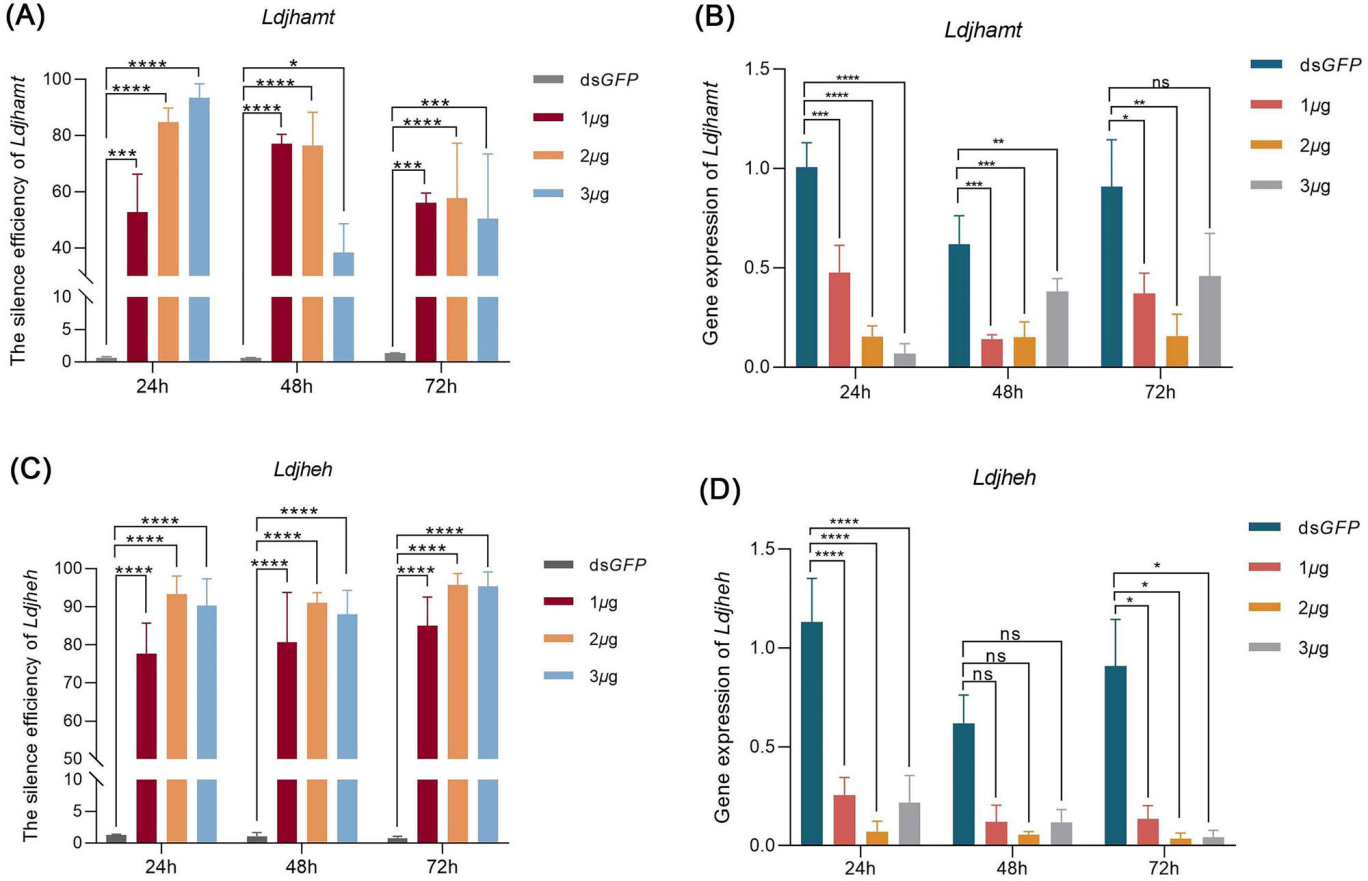
The silencing effect of RNA interference (RNAi) on target genes. (A) The silencing efficiency at different times after injecting different doses of double‐stranded (ds)*jhamt*. (B) The gene expression level of *Ldjhamt* after injection of the corresponding dose of ds*jhamt*. (C) The silencing efficiency at different times after injecting different doses of ds*jheh*. (D) The expression level of *Ldjheh* after injection of ds*jheh*. The standard errors of the means of 3 biological and 3 technical replicates are represented by error bars. In the figure, asterisks indicate significant differences in gene relative expression levels based on analysis of variance compared to the control group (*P < 0.05; **P < 0.01; ***P < 0.001; ****P < 0.0001).

Similarly, ANOVA showed that the expression level of *Ldjheh* was significantly reduced in the treated group compared to the control group. At 24 h after injection, 1, 2, and 3 *µ*g of dsRNA resulted in 77%, 94%, and 81% drop in *JHEH* mRNA levels, respectively. At 48 h after injection, the interference efficiency of 2 *µ*g dsRNA was the highest, with a 91% drop in the expression level of *JHEH* mRNA. Meanwhile, the interference efficiencies of 1 and 3 *µ*g dsRNA were 69% and 81%, respectively. Furthermore, at 72 h post‐injection, the interference efficiency continued to increase; the interference efficiency of the 2 *µ*g dsRNA remained the highest, reaching 96%. Concurrently, the interference efficiencies of the 1 and 3 *µ*g dsRNA injection groups were 85% and 95%, respectively (Fig. 2C). Similar to *Ldjhamt*, the optimal injection concentration for *Ldjheh* was 2 *µ*g. Following the injection, both the 1 and 3 *µ*g experimental groups exhibited a pattern where the target gene expression initially decreased and then increased over the 24–72 h period. In contrast, the interference efficiency of the 2 *µ*g group remained relatively stable, and did not show significant fluctuations (Fig. 2D).

### Effect of RNAi‐based silencing of Ldjhamt and Ldjheh on the development and survival of spongy moth

The effect of bioassay injections on some biological aspects of ds*jhamt* and ds*jheh* was investigated. After injecting dsRNA, we systematically monitored the developmental parameters of spongy moths under standardized rearing conditions. Comparative analysis revealed that dsRNA‐treated larvae exhibited significant reductions in body length and weight compared to the control cohort, despite identical nutritional and environmental conditions. Specifically, *Ldjhamt* knockdown demonstrated dose‐dependent phenotypic effects across larval instars. Administration of 2 *µ*g ds*jhamt* elicited the most pronounced growth inhibition, with body length reductions of 25.6% (L4), 19.5% (L5), and 25.9% (L6), respectively (Fig. 3A). Weight suppression rates followed similar patterns, with 2 *µ*g treatment achieving superior interference efficiency (56.4% in L4, 27.3% in L5, 47.1% in L6) compared to 1 *µ*g (34.8%, 14.2%, 18.9%) and 3 *µ*g (55.6%, 25.1%, 32.1%). Notably, the transient reduction in RNAi efficiency during L5 instar was followed by partial recovery in subsequent developmental stages, suggesting persistent RNAi activity (Fig. 3C). Width of larval head shell measurements paralleled growth parameter trends, with maximal width reductions observed at 2 *µ*g (L4: 26.4%, L5: 24.5%, L6: 12.6%). Lower interference efficiencies were recorded for 1 *µ*g (L4: 17.7%, L5: 14.4%, L6: 9.5%) and 3 *µ*g (L4: 20.6%, L5: 20.3%, L6: 5.6%) treatment groups (Fig. 3E).

**Fig. 3:**
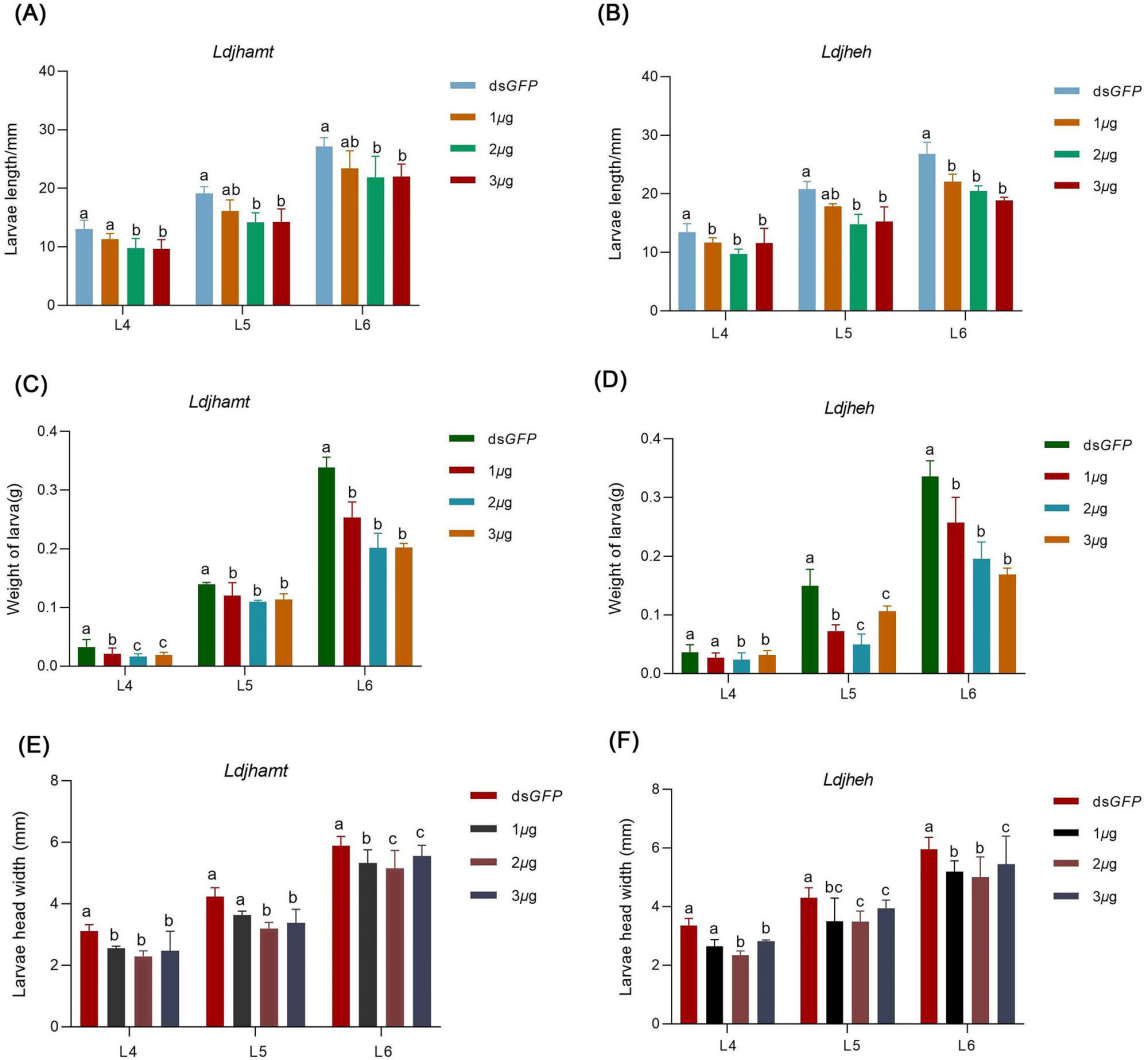
Growth and development characteristics of larvae after injection. L4, L5, and L6 representing 4th, 5th, and 6th instar larvae, respectively. (A) and (B) represent the body length of larvae at different stages after injection of double‐stranded (ds)*jhamt* and ds*jheh*, respectively. (C) and (D) represent the weight of larvae at different stages after injection of ds*jhamt* and ds*jheh*, respectively. (E) and (F) represent the head shell width of larvae at different stages after injection of ds*jhamt* and ds*jheh*, respectively. The lowercase letters indicate significant differences between treatments (*P* < 0.05, analysis of variance).

In *Ldjheh* knockdown experiments, all tested doses significantly impaired larval development. The 2 *µ*g dose demonstrated optimal interference efficacy across instars, inducing body length reductions of 27.7% (L4), 28.8% (L5), and 23.8% (L6), surpassing 1 *µ*g (L4: 13.3%, L5: 14.1%, L6: 17.6%) and 3 *µ*g (L4: 14.0%, L5: 26.6%, L6: 29.7%) treatments (Fig. 3B). Weight suppression analysis revealed dose‐responsive effects, with 2 *µ*g treatment causing 29.6% (L4), 60.7% (L5), and 37.5% (L6) reductions, significantly higher than 1 *µ*g (L4: 12.1%, L5: 35.6%, L6: 22.3%) (Fig. 3D). Width of larval head shell showed progressive reductions across treatments: 21.1%–30.4% (L4), 18.6%–18.8% (L5), and 12.9%–15.9% (L6) at 1–2 *µ*g doses, with 3 *µ*g exhibiting reduced efficiency (L4: 16.1%, L5: 8.4%, L6: 8.6%) (Fig. 3F).

The RNAi experiments targeting *JHAMT* and *JHEH* genes in *L. dispar* larvae revealed distinct developmental delays across multiple life stages. For *Ldjhamt* knockdown, dose‐dependent extension of developmental duration was observed in all post‐treatment stages. The 2 *µ*g dsRNA treatment induced most pronounced delays, increasing larval development time by 32.6% (L4), 28.9% (L5), and 42.1% (L6) compared to *GFP* controls (*P *< 0.05). This effect persisted through pupal (48.7% delay) and adult stages (38.2% delay), with developmental timelines showing progressive extension from 8.4 ± 1.1 d (ds*GFP*) to 13.8 ± 1.4 d (2 *µ*g) in pupation (Fig. 4A). *Ldjheh* silencing demonstrated stage‐specific interference patterns. While 1 *µ*g treatment caused moderate developmental prolongation in early instars (L4: 22.4% delay), maximum interference occurred at 3 *µ*g during larval‐pupal transition (29.3% pupal delay). The 2 *µ*g treatment group showed consistent interference efficiency across developmental phases, particularly in L6 (25.6% delay) and adult stages (34.1% delay) (Fig. 4B).

**Fig. 4:**
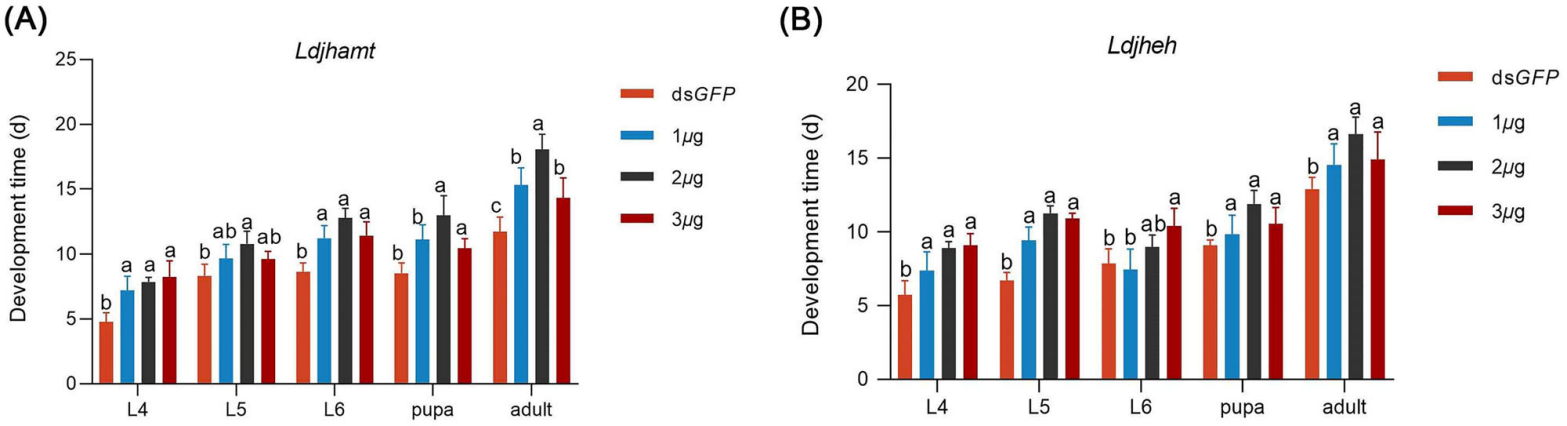
The developmental duration of *Lymantria dispar* after injection of (A) double‐stranded (ds)*jhamt* and (B) ds*jheh*. The horizontal axis represents different developmental states, with L4, L5, and L6 representing 4th, 5th, and 6th instar larvae, respectively; the vertical axis represents developmental time.

We statistically analyzed the changes in larval survival at different time points (0, 2, 4, and 6 days) after injection. The results showed that different concentrations of ds*jhamt* had an effect on larval survival compared with the ds*GFP* control group. Specifically, larval survival was significantly reduced in all dsRNA‐treated groups at 2 d after injection, and further reduced at 4 and 6 d after injection. The reductions were relatively small for the 1 and 3 *µ*g treatments and the largest reduction in the 2 *µ*g treatment. In addition, the rapid reduction in survival was concentrated in the 0–4 d period in all treatment groups (Fig. 5A). Throughout the whole observation period, the injection of ds*jheh* resulted in a decrease in larval survival compared with the control group. In particular, in the 2 *µ*g treatment group, larval survival continued to decrease rapidly from 0 to 6 d after injection, and was the lowest among the 3 treatment groups by the 6th d. The 1 *µ*g treatment was the next most effective treatment, with a lower reduction in larval survival rates from 0 to 4 d compared to the 2 *µ*g treatment group, but a rapid decrease in survival rates was observed between 4 and 6 d. The reduction in larval survival rates in the 3 *µ*g treatment group was slightly lower than that in the 1 and 2 *µ*g treatment groups, suggesting the absence of a dose‐dependent effect in the *L. dispar* RNAi experiments. Compared to injection with ds*jhamt*, ds*jheh* led to higher mortality rates (Fig. 5B). By d 6, the larval survival rate in the 2 *µ*g ds*jheh* treatment group decreased to 9.3%, compared to 33.3% in the ds*jhamt* treatment group.

**Fig. 5:**
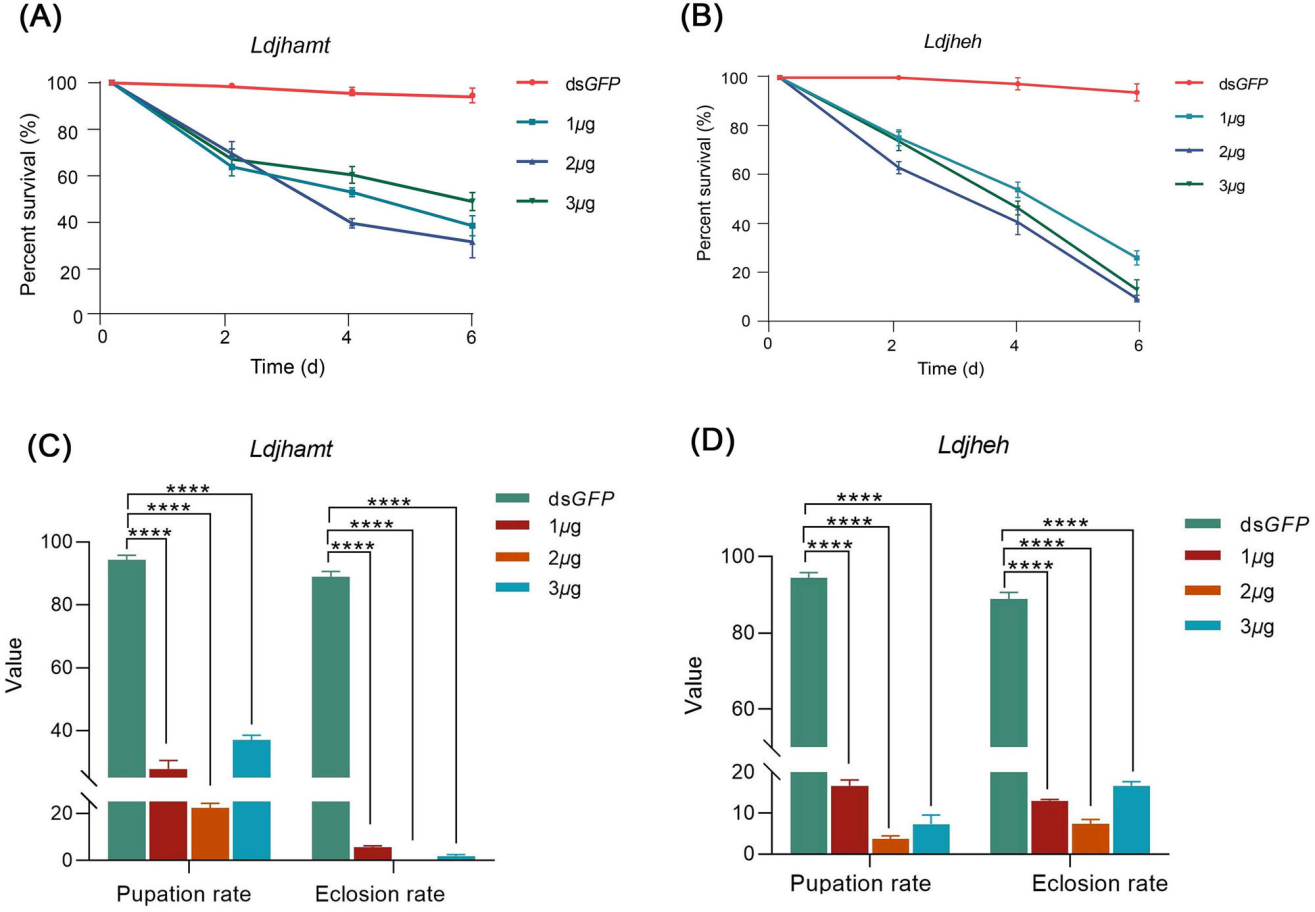
The biological effects of RNA interference (RNAi) on the growth of the spongy moth. Survival rate in larvae after the injection of double‐stranded (ds)*jhamt* (A) and ds*jheh* (B). The horizontal axis represents processing time (d), and the vertical axis represents percentage survival (%). The pupation and eclosion rates after injection of ds*jhamt* (C) and ds*jheh* (D). The horizontal axis represents the pupation rate and eclosion rate of different treatments, and the vertical axis represents the percentage (****P < 0.0001).

After ds*GFP* injection, most of the larvae successfully pupated (90.7%) and further fledged into adults. In contrast, the pupation rate was significantly lower in all dsRNA treatment groups. In terms of ds*jhamt*, the 2 *µ*g treatment group showed the most obvious inhibitory effect, with the pupation rate reduced to about 22.3%, whereas the pupation rates of the 1 and 3 *µ*g dsRNA treatment groups were reduced to about 27.8% and 37%, respectively. In terms of pupation rate, all ds*jhamt*‐treated groups showed a significant decrease, with the 2 *µ*g treatment group showing the lowest rate of 22.30%, while the adult eclosion rates were reduced to 5.6%, 0%, and 1.9%, respectively (Fig. 5C). Similarly, all ds*jheh* treatment groups also significantly reduced the pupation and adult eclosion rates of spongy moth. The pupation rates of the 3 treatment groups were 16.7%, 3.7%, and 7.4%, while the adult eclosion rates were reduced to 13%, 7%, and 17%, respectively (Fig. 5D). These results were statistically analyzed to be significantly different (****P < 0.0001).

We further observed the morphological characteristics of the RNAi‐treated group and the control group at different developmental stages; the injected larvae showed molting failure compared to the control group, regardless of whether they were injected with ds*jhamt* or ds*jheh*. Specifically, the new epidermis had formed but the old epidermis still existed. Similarly, the pupae also showed developmental malformations, with malformed individuals showing incomplete transition from larva to pupa, with the head in larval form and the abdomen in pupa form, and the color of malformed pupae was darker, while the color of normally developed pupae was reddish brown. In addition, compared with normal adults, target knockout adults showed smaller wings and abnormal morphology, and the morphologically abnormal adults were unable to mate and died within 3 d after emergence (Figs. 6 and 7).

**Fig. 6:**
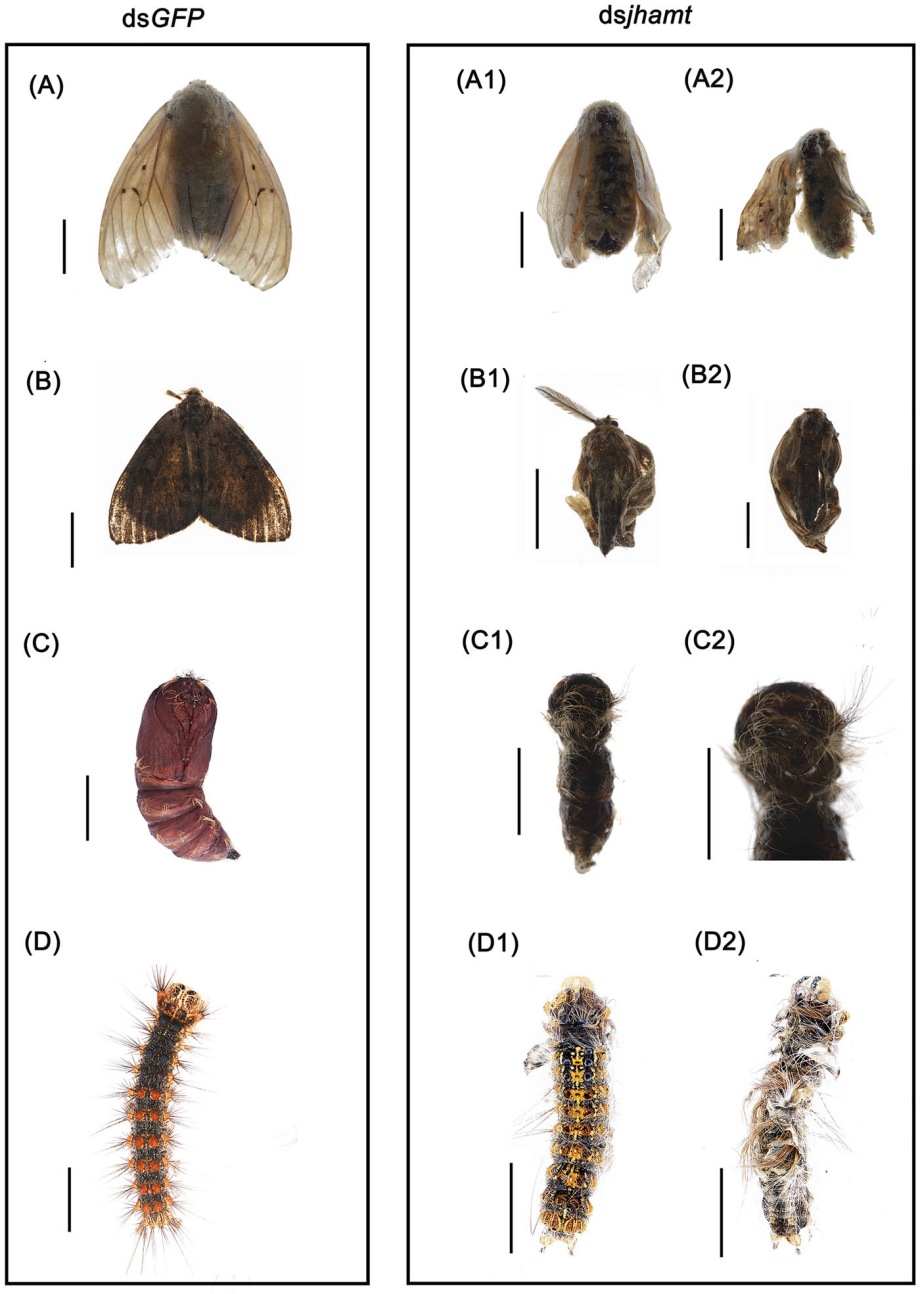
Compared with injection of double‐stranded (ds)*GFP*, injection of ds*jhamt* resulted in morphological changes (scale bar = 1 cm) in the larvae, pupae and adults of the spongy moth. (A, B, C and D) are the normal developmental spongy moth in the control group. (A1–D2) are the developmental abnormalities caused by injection of ds*jhamt*.

**Fig. 7:**
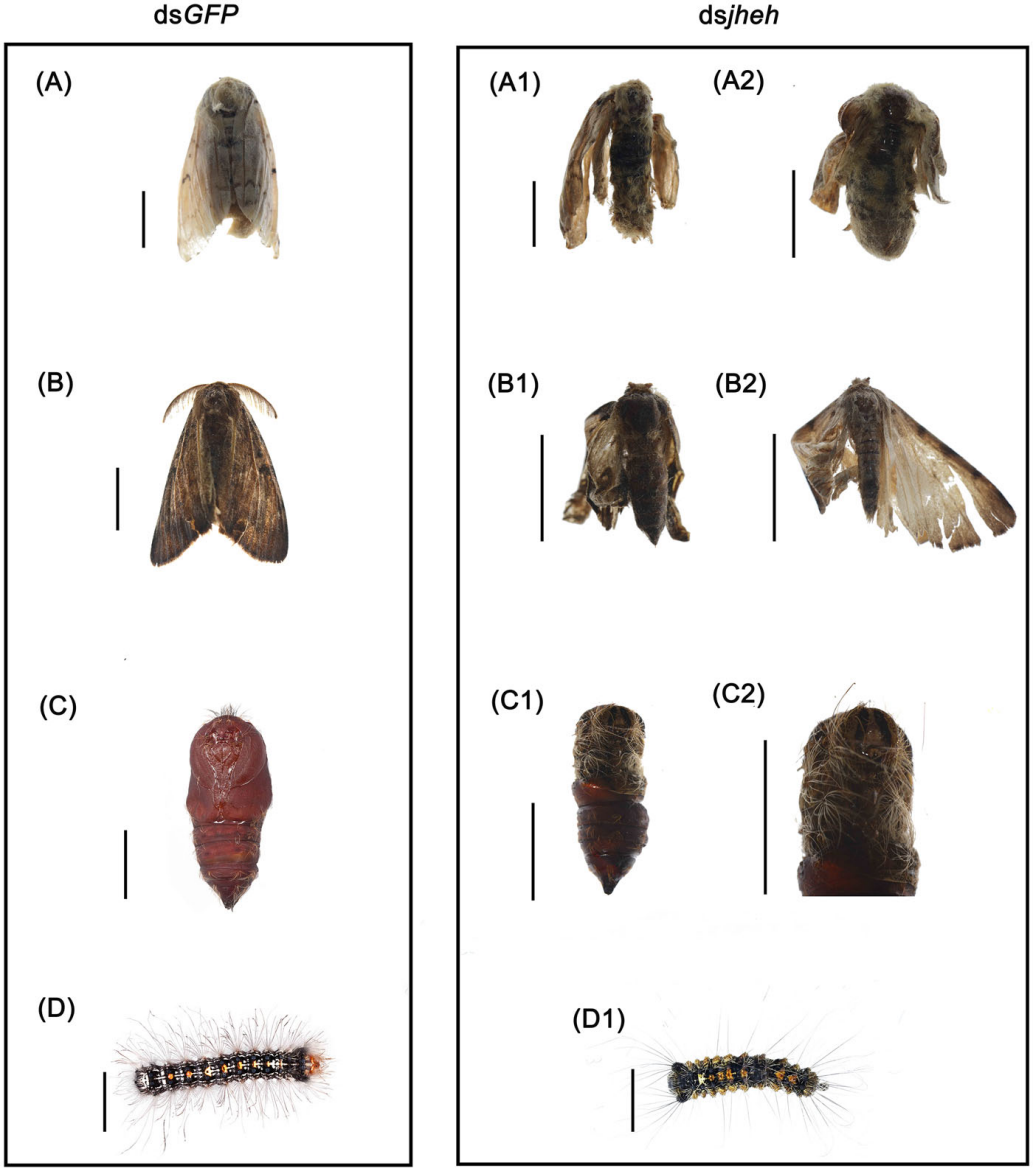
Compared with injection of double‐stranded (ds)*GFP*, injection of ds*jheh* resulted in morphological changes (scale bar = 1 cm) in the larvae, pupae, and adults of the spongy moth. (A, B, C, and D) are the normal developmental spongy moth in the control group. (A1–D1) are the developmental abnormalities caused by injection of ds*jheh*.

## Discussion

In insects, JH and 20‐hydroxyecdysone (20E) play indispensable roles in embryo development. Among these, 20E triggers continuous molting, while JH regulates metamorphosis by maintaining larval growth and initiating metamorphosis. Thus, JH titers are tightly regulated through their precise synthesis and degradation at different stages of insect development (Hammock, 1985). During the larval stage, high‐droplet JH promotes molting and larval (or nymph) growth. Further, during the transition of larva to pupa, JH titers decrease significantly, while 20E titers increase (Truman & Riddiford, 2007). JHAMT is a key enzyme involved in the JH biosynthetic pathway, and it plays an important role in regulating various developmental and physiological processes in insects. JHEH is an even more critical enzyme involved in irreversibly hydrolyzing JH to its diol product and shows great potential in controlling the insect population (Swetha 2015). Thus, these genes were chosen in this study mainly based on their critical roles in regulating insect physiology and development. The present research silenced *jhamt* and *jheh* genes using the RNAi approach to assess their significance as targets for pest control strategies (Swetha 2015).

RNAi, a powerful reverse genetic tool, is widely used in studying gene functions (Kunte *et al.*, 2020). This approach has also been adopted in pest control. However, it has been shown that when dsRNA is fed to spongy moth larvae, the expression of the target genes does not decrease significantly within 72 h due to the degradation of dsRNA. Moreover, researchers found that the ingesting method and the dsRNA concentration had no significant effect on the survival and phenotype of the larvae (Wen *et al.*, 2018). To overcome this challenge and assess the potential of novel control strategies based on RNAi, we screened *Ldjhamt* and *Ldjheh* from the spongy moth transcriptome database for in‐depth analysis. We first successfully cloned the full‐length *Ldjhamt* and *Ldjheh* genes and analyzed these sequences. We also found that the proteins encoded by the *jhamt* gene were mainly located in the cytoplasm and did not possess signal peptide sequences, consistent with earlier reports (Sun *et al.*, 2022). Studies have shown that the N‐terminus of the JHEH protein contains a transmembrane structural domain in a variety of insects such as *Manduca sexta* (Grieneisen *et al.*, 1995), *Trichoplusia ni* (VanHook Harris *et al.*, 1999), *Ctenocephalides felis* (Keiser *et al.*, 2002), and *Leptinotarsa decemlineata* (Lü *et al.*, 2015). Consistent with these earlier reports, we found a transmembrane structural domain at the N‐terminus of the *Ld*JHEH protein of the spongy moth.

Further, a highly sensitive RT‐qPCR technique was adopted to systematically assess the dynamic changes in the expression of *Ldjhamt* and *Ldjheh* during the development of spongy moth. Previous studies have shown that the temporal expression pattern of *jhamt* in Clostera anastomosis is closely related to the fluctuations in JH titers in the hemolymph. Previous studies have found that *Bmjhamt* expression was high from the 1st to the penultimate instar and rapidly declined in the early terminal instar, revealing a close link between the transcriptional activity of *jhamt* at the end of larval life and the metamorphosis of larva to pupa (Shinoda & Itoyama, 2003). The present study found that the expression level of *Ldjhamt* in the egg, pupa, and adult of the spongy moth was significantly lower than that in the larva, and the trend of *Ldjhamt* expression at the larval stage was similar to that of *jhamt* in *Dendroctonus armandi*. The low expression of *Ldjhamt* in pupa and adults strongly supports the hypothesis that *Ldjhamt* plays a key role in determining the time point of larval to pupa metamorphosis by regulating the rate of JH biosynthesis.

On the other hand, *Ldjheh* was expressed in the spongy moth at an every stage of development, but its expression decreased gradually from the larval to the adult stage and peaked at the 6th instar stage. Similarly, the highest expression of *jheh* was observed in the last 1st instar after ds*jheh* injection into potato beetles (Lü *et al.*, 2015). In addition, the present study found high expression of both *Ldjhamt* and *Ldjheh* in fat bodies; this demonstrates that the fat body has an important role in the regulation of JH metabolism. These findings suggest that the fat body is important in regulating JH metabolism. Compared with *jhamt*, *jheh* was also expressed in the egg, pupa, and adult of the spongy moth, probably due to the extensive involvement of JH in regulating several physiological processes such as insect development and diapause. Our analysis proved this significance and suggested their potential in pest control.

Studies have indicated that the efficiency of silencing using dsRNA is low in Lepidoptera due to various factors (Kim & Zhang, 2022), such as the alkaline environment of the midgut. Generally, RNA is susceptible to hydrolysis in high‐pH environments, reducing gene silencing efficiency (Bernhardt & Tate, 2012). Initial analysis showed peak expression of *jhamt* and *jheh* in the last instar larvae. A significant decrease in the expression of the target genes was observed at 24 h after the ds*jhamt* injection. Still, a rebound in the expression level was observed at 72 h after the injection, decreasing the interference efficiency. Despite this rebound phenomenon, by continuously monitoring larval growth and development after injection, we found that injection of dsRNA targeting 2 target genes resulted in significant reductions in larval body length, body weight, and cephalic carapace width compared to controls. Disruption of both the RNAi treatments *Ldjhamt* and *Ldjheh* significantly inhibited larval length development of *Lymantria dispar* larvae and this inhibition was seen throughout larval growth. The inhibitory effect lasted throughout the entire larval growth stage. In addition, the molting interval of larvae of different instars, as well as the development time required for pupae and adults, also significantly increased.

Further counting the larval mortality, we found that most larvae died within 0–4 d. For *Ldjheh*, from d 0 to d 6, we observed that the pupation and eclosion rates of the experimental group larvae were significantly lower than those of the control group, indicating that dsRNA treatment effectively interfered with the normal developmental process of the larvae. In particular, larvae treated with 2 *µ*g ds*jhamt* showed the lowest eclosion rate, dropping to 0, which further emphasizes the critical impact of RNAi on the development of moths. It is worth noting that although there was a certain degree of rebound in the expression level of target genes after RNAi treatment, this rebound did not seem to completely reverse the effect of RNAi. Our research results indicate that dsRNA treatment has a sustained impact on larvae, leading to their death or developmental failure, such as interruption of pupation and eclosion processes.

In contrast, we observed a sustained decline in the expression of the target gene with no rebound between 24 and 72 h after ds*jheh* injection. This observation suggests that dsRNA was not significantly degraded during this 72 h period. This result fully demonstrates the potential that the *Ldjheh* gene can be used as a novel preventive tool for the spongy moth. Detailed analysis showed that both ds*jhamt* and ds*jheh* injections resulted in the death of numerous larvae due to the inability to shed the old epidermis. The residual epidermis on the larval appendages severely interfered with their feeding and crawling abilities, which eventually led to death. It should be noted that the death of these larvae is not directly related to the trauma caused by microinjection, as we observed that the majority of larvae in the control group successfully pupated (90.7%) and emerged, indicating that the trauma caused by injection does not seriously affect the normal growth and development of the moth. In addition, in a functional analysis of the heat shock protein *hsp40* gene mediated by RNAi technology, the experimental group and the control group did not show significant differences in key physiological indicators such as relative growth rate, food utilization rate, food conversion rate and approximate digestibility (Wang & Wen, 2014). This indicates that although the injection process may cause some trauma, the larvae of the spongy moth can adapt to this trauma through their own physiological regulatory mechanisms, without affecting their overall growth and development. Furthermore, RNAi‐treated specimens exhibited developmental aberrations across both pupal and adult stages. Affected individuals displayed incomplete larval‐pupal metamorphosis, characterized by developmental arrest during critical transitional phases. Adult phenotypes manifested severe wing hypoplasia and impaired emergence. A similar trend was found with the injection of ds*jheh* in *Nilaparvata lugens*. From the 3rd to 7th d after injecting the ds*Nljhe* + ds*Nljheh* mixture, the survival rate was significantly reduced, and all injected nymphs died from abnormal molting (Zhao *et al.*, 2017). In *Apolygus lucorum*, the survival rate of the dsRNA‐*Alucjheh* injected group was significantly reduced from 2 d after injection compared to the control and ds*GFP*‐injected groups. In addition, individuals injected with dsRNA‐*Alucjheh* exhibited molting difficulties and eventually died. Although the dissolution and sliding of the old cuticle were observed, the nymphs were found trapped in the old cuticle and died (Tusun *et al.*, 2017). These results confirm that by specifically silencing target genes related to juvenile preserving hormones in insects significantly affects their development. Moreover, most of the treated insects exhibited larval molt failure leading to death, confirming the role of juvenile preserving hormones in the regulation of growth and development in insects.

Through a series of dsRNA injection experiments with different concentration gradients, we observed that in spongy moth, it is not the higher the injection concentration that leads to more significant gene silencing efficiency. Specifically, both for *Ldjhamt* and *Ldjheh*, the mRNA interference efficiency in the high dsRNA concentration group (3 *µ*g) was lower than that in the low concentration groups (1 and 2 *µ*g). Thus, we found that the injection of 2 *µ*g of dsRNA resulted in the highest interference efficiency of the target gene, and this concentration of dsRNA resulted in the highest mortality rate of spongy moth. Therefore, we suggest that 2 *µ*g can be used when utilizing RNAi‐mediated targeting of *Ldjhamt* and *Ldjheh* for spongy moth control.

RNAi technology, with high specificity, effectively reduces potential harm to non‐target and off‐target species and has long‐lasting protective effects (de Andrade & Hunter, 2016; Niu *et al.*, 2023). Compared to traditional chemical pesticides, RNAi‐based pest management techniques possess significant advantages. *L. dispar* is a globally important quarantine pest that has caused severe damage to numerous forest tree species and is considered a global threat to commercial and native forest systems (Peterson *et al.*, 2007). Considering the wide use of chemical pesticides in controlling spongy moths and its well‐known limitations (Wanner *et al.*, 2002; Papadopoulou *et al.*, 2012), we evaluated the potential of RNAi technology in controlling this pest. Studies have indicated that the unique pH environment and the specific RNA nuclease in the intestine of lepidopteran pests lead to a less stable, disrupting effect of RNAi than coleopteran pests. Fortunately, silencing *jhamt* and *jheh* using dsRNA resulted in significant mortality and molt‐related morphological abnormalities in the spongy moth. Specifically, ds*jheh* injection led to higher mortality than ds*jhamt*, and the expression level of *Ldjheh* was more significantly inhibited. In addition, the injection method showed significant results in inducing mortality compared to oral delivery. However, the injection method does face many challenges in practical applications, such as the need for specialized equipment and operating techniques, and the time‐consuming and labor‐intensive process of injecting each insect individually. But it is of great significance in basic research and exploratory applications. Injection method is currently one of the most effective delivery methods in RNAi experiments, and it is also an important tool in insect molecular biology research, especially widely used in insect gene function research. Injection can directly deliver dsRNA into insect bodies, rapidly verifying gene function. Through injection, we can efficiently silence target genes and observe their effects on insect growth, development, behavior and physiological processes. In future research, the shortcomings of the injection method can be improved through the following approach. (1) Optimizing the efficiency of RNAi by improving the delivery system: studies have shown that the combination of nanotechnology and RNAi has great potential to become a more sustainable pest management strategy (Yan *et al.*, 2021; Zhang *et al.*, 2022). Chitosan/dsRNA‐based nanoparticles can cause significant mortality, delayed growth and development and lead to wing deformities in adult *Aedes aegypti* mosquitoes (Kumar *et al.*, 2016). (2) Non‐invasive delivery method: by spraying preparations containing dsRNA or through microbial mediated delivery, some studies have reported that spray/topical application of dsRNA/siRNA preparations penetrate into the insect cuticle to induce lethal effect (Zhang *et al.*, 2015; Dias *et al.*, 2019).

## Conclusion

In conclusion, for the major quarantine pest *L. dispar*, we used an injection method to specifically silence *Ldjhamt* and *Ldjheh*, which are related to the JH, in addition to causing a significant decrease in the expression levels of target genes, we found that the injection of the dsRNA of the target genes led to a large number of deaths of spongy moth, which was not found in the control group. Subsequently, a significant decrease in growth and developmental indicators (larval body length, larval body weight, and head capsule width), a significant increase in developmental time at different developmental stages, and a significant decrease in pupation and eclosion rates were observed in the treated group. This study confirms the potential application of RNAi technology in spongy moth control and demonstrates the potential of *Ldjhamt* and *Ldjheh* as target genes. This study provides novel insights into the key genes regulating spongy moth development and opens avenues for exploring control strategies for this pest. This finding will facilitate the optimization of pest management and the design and development of novel bioinsecticides.

## Disclosure

The authors have no relevant financial or non‐financial interests to disclose.
